# Multimodal imaging of cubic Cu_2_O@Au nanocage formation via galvanic replacement using X-ray ptychography and nano diffraction

**DOI:** 10.1038/s41598-022-26877-6

**Published:** 2023-01-06

**Authors:** Lukas Grote, Sarah-Alexandra Hussak, Leif Albers, Karolina Stachnik, Federica Mancini, Martin Seyrich, Olga Vasylieva, Dennis Brückner, Mikhail Lyubomirskiy, Christian G. Schroer, Dorota Koziej

**Affiliations:** 1 Center for Hybrid Nanostructures, Institute for Nanostructure and Solid-State Physics, University of Hamburg, Luruper Chaussee 149, 22761 Hamburg, Germany; 2 Center for X-ray and Nano Science CXNS, Deutsches Elektronen-Synchrotron DESY, Notkestraße 85, 22607 Hamburg, Germany; 3 Deutsches Elektronen-Synchrotron DESY, Notkestraße 85, 22607 Hamburg, Germany; 4 Institute of Science and Technology for Ceramics (ISTEC), National Research Council (CNR), Via Granarolo 64, 48018 Faenza, RA Italy; 5 Helmholtz Imaging Platform, Deutsches Elektronen-Synchrotron DESY, Notkestraße 85, 22607 Hamburg, Germany; 6 The Hamburg Center for Ultrafast Imaging, Hamburg, Germany

**Keywords:** Nanoscale materials, Imaging techniques, Materials science, Characterization and analytical techniques, Chemical physics, Imaging techniques

## Abstract

Being able to observe the formation of multi-material nanostructures in situ, simultaneously from a morphological and crystallographic perspective, is a challenging task. Yet, this is essential for the fabrication of nanomaterials with well-controlled composition exposing the most active crystallographic surfaces, as required for highly active catalysts in energy applications. To demonstrate how X-ray ptychography can be combined with scanning nanoprobe diffraction to realize multimodal imaging, we study growing Cu_2_O nanocubes and their transformation into Au nanocages. During the growth of nanocubes at a temperature of 138 °C, we measure the crystal structure of an individual nanoparticle and determine the presence of (100) crystallographic facets at its surface. We subsequently visualize the transformation of Cu_2_O into Au nanocages by galvanic replacement. The nanocubes interior homogeneously dissolves while smaller Au particles grow on their surface and later coalesce to form porous nanocages. We finally determine the amount of radiation damage making use of the quantitative phase images. We find that both the total surface dose as well as the dose rate imparted by the X-ray beam trigger additional deposition of Au onto the nanocages. Our multimodal approach can benefit in-solution imaging of multi-material nanostructures in many related fields.

## Introduction

In the chemistry of nanomaterials, the evolution of nanoparticle shape and morphology during the synthesis is equally important as the exposure of the most active crystallographic surfaces in the course of crystallization. This way, nanomaterials with an optimized shape and crystallinity can reach highest performance in catalytic applications, such as (photo)-electrochemical water splitting^1–3^ and CO_2_ reduction^4,5^.

Since the first discoveries of nanostructured materials^6,7^, high-resolution transmission electron microscopy (HR-TEM) has been employed to make a connection between their shape and crystallinity^8,9^. Due to its unrivaled spatial resolution, HR-TEM soon uncovered the adaption of the outer shape of nanoparticles to the underlying crystal system, and the exposure of specific low-energy crystallographic planes at the particles’ facets soon became evident^10–13^. Later, the lattice orientation of individual nanocrystals could be visualized with electron diffraction^14–16^. However, to gain rational control over a synthesis, in situ visualization of the shape and crystal structure of nanoparticles during formation and transformation processes is a decisive yet difficult task.

The applicability of electron microscopy for in situ monitoring is limited since the liquid environment of a chemical synthesis is often opaque for electrons. Hard X-rays, on the other hand, can penetrate thick sample environments and are thus an ideal probe for in situ microscopy^17^. To this end, X-ray ptychography is a versatile and convenient method. It is a scanning coherent imaging technique allowing to reconstruct transmission images of a sample from a series of small-angle diffraction patterns^18–26^. When imaging nanostructures with similar thicknesses, the phase images allow to roughly distinguish materials with different densities, making the method especially powerful to follow the interactions and individual shape evolutions of different compounds in a multi-material nanostructure. When imaging ex situ with low scattering background, a spatial resolution of 10 nm can be reached^27,28^. In our previous work, we showed that hard X-ray ptychography is robust enough to image weakly scattering nanoparticles inside a liquid chemical reactor with a thickness of 1 mm, while still achieving a resolution of 66 nm^29^.

Scanning wide-angle X-ray diffraction (WAXS) in the nanofocused X-ray beam complements the ptychographic images by imaging the crystallinity of the nanomaterial^30–34^. In this way, grain rotation and lattice deformation in AgBr crystals^35^ as well as the orientation of crystalline domains in a single CsPbBr_3_ perovskite nanowire^36^ were visualized.

**Figure 1:**
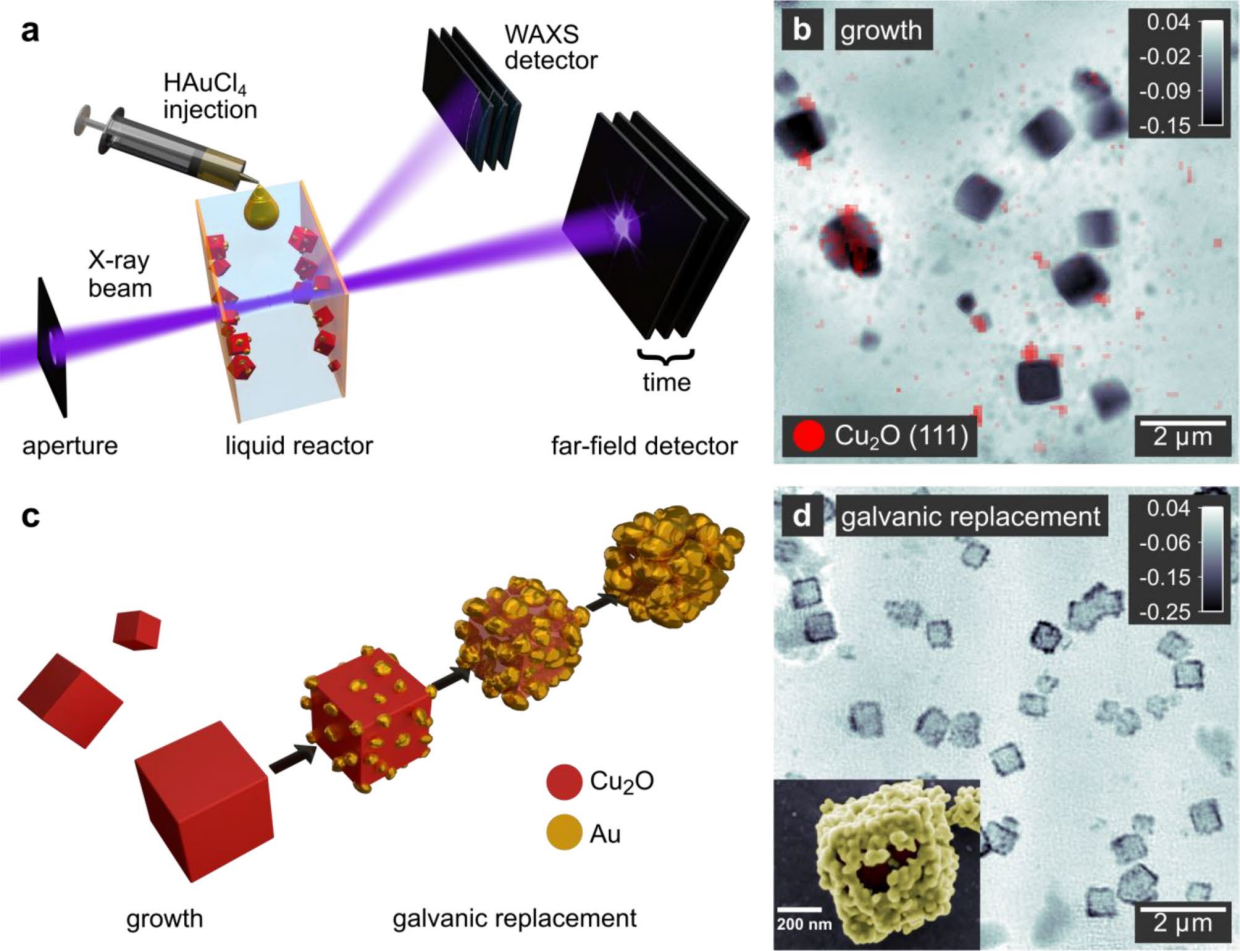
Outline of the multimodal in situ imaging experiment on Cu_2_O@Au nanocages. (**a**) Schematic illustration of the experimental setup showing a section of the in situ reactor for simplicity. The nanocubes grow on the inner walls of the reactor. (**b**) Ptychographic reconstruction of Cu_2_O nanocubes growing inside the chemical reactor. The image is overlaid with the spatially resolved diffraction signal (in red), representing diffraction from the Cu_2_O (111) crystallographic plane. (**c**) Illustration of the observed reaction pathway including the growth of Cu_2_O nanocubes and their morphological transformation into gold nanocages during the galvanic replacement. (**d**) In situ ptychographic reconstruction of the nanocubes undergoing an electroless galvanic replacement reaction with gold. The gray scale indicates the phase shift in radian. Inset: False-colored SEM image of a partly replaced nanocube.

Here, we extend the capabilities of in-solution imaging by combining simultaneously hard X-ray ptychography with scanning nano WAXS during the solvothermal growth of cuprous oxide (Cu_2_O) nanocubes (Fig. 1a). We obtain highly spatially resolved crystallographic information directly overlaid with the morphological characterization in the ptychographic images, allowing us to identify the shape as well as the crystal structure and lattice orientation of a single nanocube in situ (Fig. 1b).

In a second step, we use the Cu_2_O nanocubes as a template in a galvanic replacement reaction (GRR) with HAuCl_4_ (Fig. 1c). Galvanic replacement represents a versatile route to a variety of multi-material and hollow nanostructures^37,38^. In the case of metal oxide nanoparticles smaller than 100 nm, voids forming in the particle interior during the GRR were observed^39,40^, which were accounted to a pinhole corrosion mechanism upon dissolution of the core^41–43^.

In this study, we combine the material contrast of the ptychographic phase images with WAXS to spot isolated Au nanoparticles nucleating on the surface of the nanocubes while the Cu_2_O core is oxidized and dissolves (Fig. 1d). In the course of the GRR, we observe the Au nanoparticles growing and coalescing into nanocages. Furthermore, the projection images allow us to follow structural changes of the interior of the nanocubes with sizes up to 1 μm.

Finally, the phase shift in the ptychographic images allows to quantify the amount of material^44^. This makes it possible to follow the progress of the chemical transformation, and at the same time to determine the occurrence of radiation damage. We find that the interaction with the X-ray beam triggers additional Au deposition compared to a GRR without exposure to X-rays. We show that a surface dose^45^ of 2.1 MGy required for a single image does not cause visible radiation damage, however, as the dose of subsequent image acquisitions accumulates, radiation damage becomes significant. An experiment with a more intense X-ray beam shows that a dose rate higher than 18.1 MGy/s in the X-ray focus strongly amplifies the effect.

## Results and discussion

**Figure 2:**
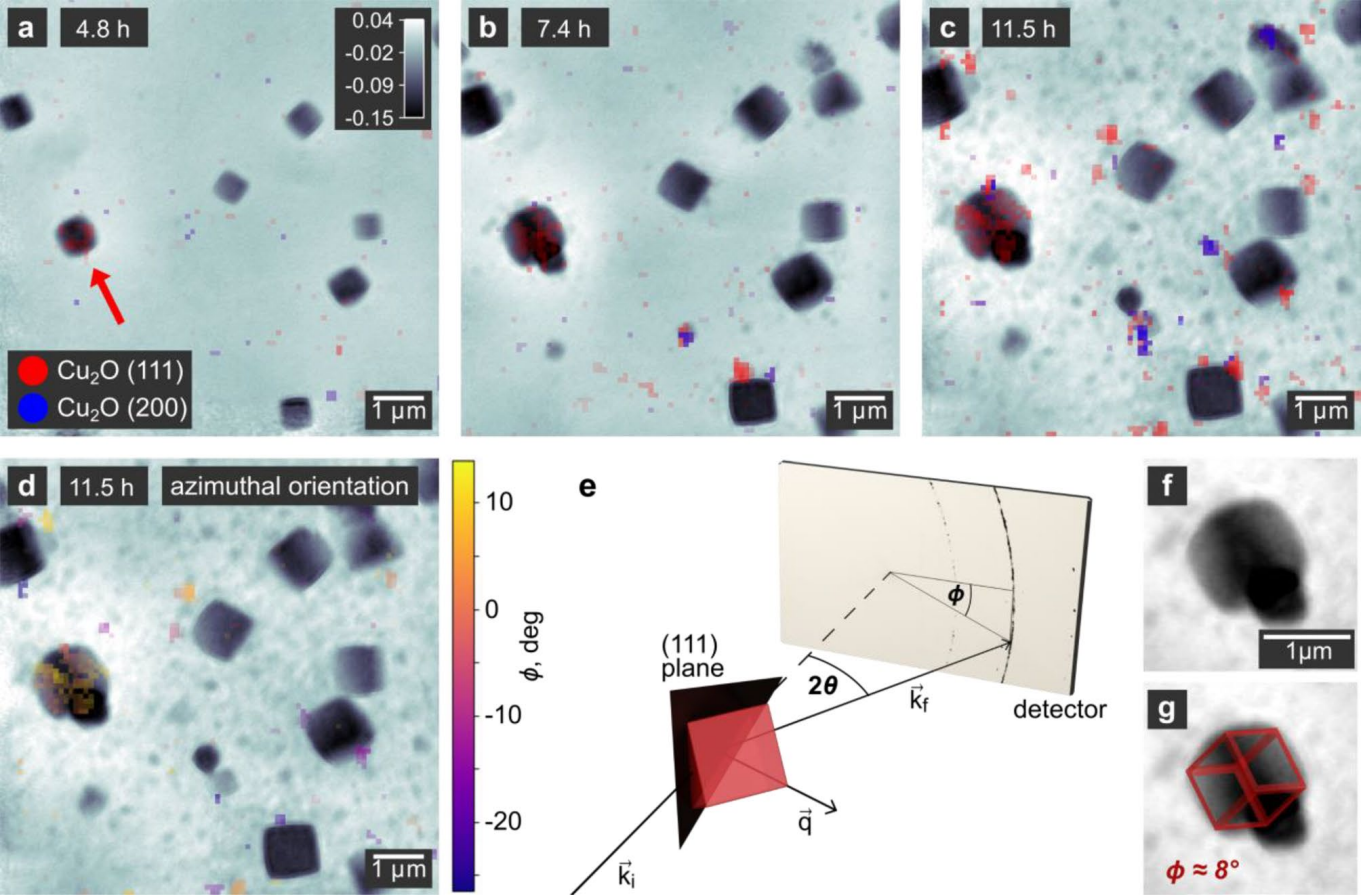
Crystallinity of growing Cu_2_O nanocubes. (**a**–**c**) In situ ptychographic reconstructions during the growth of the Cu_2_O nanocubes overlaid with the spatially resolved nano-diffraction signal. The crystallographic orientation is highlighted by color, whereas the gray scale indicates the phase shift of the object in radian as obtained by ptychography. While most of the single-crystalline nanocubes are oriented in a way not fulfilling the Bragg condition, the particle marked with an arrow diffracts at the (111) plane. (**d**) Same ptychographic reconstruction as in **c**, but here the overlay indicates the azimuthal orientation of the (111) crystallographic plane. The highlighted particle exhibits an azimuthal rotation of $\rm{{\phi}}\approx 8^{\circ}$. (**e**) Schematic illustration of the single-particle diffraction geometry showing the incident beam $\overrightarrow{{k}_{i}}$, the diffracted beam $\overrightarrow{{k}_{f}}$ and the scattering vector $\overrightarrow{q}$. It allows to estimate the particle orientation from the position of its (111) reflection. The rotation angle around the normal of the crystallographic plane remains undefined. (**f**) Magnification of the nanocube highlighted in (**a**–**d**). In (**g**), this particle is shown together with a schematic cube representing one possible orientation determined from the diffraction experiment. The orientation of the cubic Cu_2_O crystal lattice matches that of the cubic particle.

The reaction of copper acetylacetonate with benzyl alcohol is a versatile route which enables the synthesis of different Cu and Cu_2_O nanoparticle morphologies^46,47^. In our previous work, we used X-ray ptychography to follow the growth of Cu_2_O nanocubes in a liquid reactor, demonstrating the applicability of X-ray microscopy for in situ observations of the growth of nanomaterials^29^. However, the questions about the crystallinity and the exposure of specific crystallographic facets at the surface of the nanocubes remained unanswered. To this end, we designed an advanced experiment and combine ptychographic imaging with simultaneous scanning WAXS. The ptychographic reconstructions can then be overlaid with the additional contrast, resulting in multimodal microscopic images. Here, using the same example of Cu_2_O nanocubes, we show an extended microscopy experiment, following the crystallization and the orientation of the crystal lattice of an individual nanocube (Fig. 2).

### Linking crystallization and shape during growth of nanocubes

To synthesize Cu_2_O nanocubes^47,48^, we fill the in situ reactor with precursor solution and heat it to 138 °C for 19 h. Detailed information on the reactor can be found elsewhere^29^, as well as in Supplementary Note 1. The nanocubes have a strong affinity to the poly-imide walls of the reactor^29^, thus most of them nucleate and grow on both windows. It takes 26 min to acquire a ptychographic image, but the particles remain immobilized at the polyimide even during a series of images.

As the nanocubes grow on both the entrance and the exit windows of the reactor, an X-ray projection image would usually show contributions from these two layers of particles. However, ptychography allows to retrieve several phase images at different positions along the optical axis. This procedure is called multi-slicing^49,50^, and we use it to reconstruct separate images of nanocubes on the two windows^17^. Due to a slow deformation of the window material, the distance between them decreased during the time series, which we took into account for the multi-sliced reconstructions (Supplementary Fig. 2).

A typical WAXS pattern from a single scan point is shown in Supplementary Fig. 3a. Since the X-ray focus is smaller than an individual nanocube, most patterns show either none or a single reflection from an individual nanocube (inset of Supplementary Fig. 3a). The background-subtracted sum of all WAXS patterns acquired along a full ptychographic scan (Supplementary Fig. 3b) typically shows less than five reflections, indicating that only a small fraction of the particles fulfill the diffraction condition.

The distance between the windows results in a small offset of the scattering angle in the WAXS measurement for the two particle layers, which we use to generate separate WAXS intensity distributions from the two windows (for details, see Methods section and Supplementary Fig. 4).

Figure 2a–c show ptychographic images overlaid with spatially resolved WAXS distributions taken during the growth of the nanocubes. After 11.5 h, the nanocubes reach their final size. While the population of large particles just below 1 μm in size dominates, also smaller nanocubes are visible. We estimate the spatial resolution of the ptychographic images using Fourier ring correlations (FRC)^51^, which we obtain by splitting the diffraction patterns of a single ptychogram into two separate sets. We then reconstruct the sets individually and calculate the FRC of the two resulting images (cf. Supplementary Note 2 and Supplementary Fig. 5). Applying the half-bit criterion^52^ to compensate for the quality loss introduced by splitting the data set, we find a spatial resolution of about 104 nm. While the spatial resolution in ptychography is independent of the focus size, the latter defines the resolution of the scanning WAXS measurement. As it can be seen from the beam profile in Supplementary Fig. 6, only the exit window is positioned in the X-ray focus with a size of 135 nm horizontally by 207 nm vertically. Thus, only images taken on the exit window of the reactor have an optimal WAXS resolution.

We focus on the Cu_2_O (111) and (100) reflections to study the lattice orientation of individual nanocubes in relation to their shape. The nanocube highlighted with a red arrow in Fig. 2a exhibits the Cu_2_O (111) reflection, confirming the formation of the expected crystal structure. At later growth stages, almost the entire area of this nanocube shows the same reflection, indicating that this nanocube is a single crystal. After 11.5 h (Fig. 2c), the WAXS image even resembles the cubic shape of the particle visible in ptychography. We observe that no other nanocube in the field of view exhibits a clear reflection, which further supports the single-crystalline nature of the particles. In Fig. 2c,d, we also observe WAXS signal without a corresponding nanocube in the ptychographic images, which we account to an incomplete separation of the WAXS signals from the entrance and exit windows (see Supplementary Fig. 4), as well as to particles dispersed in the solution.

The orientation of the scattering vector $\overrightarrow{q}$ with respect to the incident X-ray beam $\overrightarrow{{k}_{i}}$ has a polar component $\theta$ corresponding to the diffraction angle, and an azimuthal component $\phi$ (Fig. 2e). Figure 2d shows the spatial distribution of the azimuthal component. Again, only the highlighted nanocube gives a diffraction signal which is consistent over its entire area. On average over the in situ time series, the nanocube has an azimuthal orientation of $\phi \approx 8^\circ$. Together with the known diffraction angle of $2{\theta }_{111}=19.31^\circ$ at 15 keV, we determine the orientation of the cubic Cu_2_O crystal lattice and schematically overlay it with the outer shape of the nanocube (Fig. 2f,g). Although the lattice orientation orthogonal to $\overrightarrow{q}$ remains indefinite in this measurement, we find an orientation of the lattice matching the visible orientation of the cubic particle. We can thus conclude that during its entire growth, the nanocube exhibits (100) crystallographic planes at its surfaces.

### Cu_2_O@Au nanocages: phase imaging differentiates materials and their respective morphologies

In a next step, we transform the as-prepared Cu_2_O nanocubes in an electroless galvanic replacement process with HAuCl_4_ into porous Au nanocages. The reaction follows Eq. (1)^43^.

1 $$1.5 {\text{Cu}}_{2}{{\text{O}}}_{(s)}+{\text{AuCl}}_{4 (aq)}^{-}+3 {\text{H}}_{(aq)}^{+}\to \text{Au}_{(s)}+3 \text{Cu}_{(aq)}^{2+}+4 \text{Cl}_{(aq)}^{-}+1.5 {\text{H}}_{2}\text{O}$$

In the case of polyhedrally shaped Cu_2_O templates, previous studies found that corners and edges get covered with Au before the facets^53–55^. Also, Au preferentially adsorbes to (111) facets first before covering (100) facets in response to the difference in surface energy^56^. However, in the case of cubic templates exhibiting only (100) facets, we can expect a uniform growth of the Au layer. Furthermore, the pH of the reaction medium has a strong effect on the morphology of the resulting Au layer^53^. While an acidic medium increases the nucleation efficiency of Au and leads to a smooth layer, a less acidic pH favors particle-like and grainy Au deposition. In our case, to protect the Cu_2_O from being etched during the imaging experiment, we use the reactants at neutral pH.

To start the replacement of Cu_2_O with Au, we mount one window with nanocubes at the exit position of the reactor together with one empty window and pre-fill the reactor with water. We slowly dose the reaction solution into the reactor and image the gradual replacement during 10 h.

**Figure 3:**
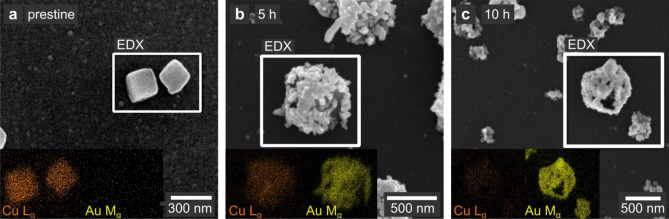
Ex situ study of the galvanic replacement of Cu_2_O with Au. SEM images and corresponding EDX maps (insets) of (**a**) pristine and (**b**,**c**) Cu_2_O nanocubes replaced with HAuCl_4_ for 5 and 10 h, respectively. After 5 h (**b**), the GRR is still incomplete, with a grainy layer of Au particles growing at the surface of Cu_2_O. After 10 h (**c**), the GRR is complete, yielding hollow Au nanocages.

The scanning electron microscopy (SEM) images in Fig. 3 depict the changes in the particle morphology during the GRR. Even though the size of the nanocubes varies between these ex situ experiments, we observe the deposition of individual Au particles with different shapes on their surface. While after 5 h reaction time (Fig. 3b), the Cu_2_O template underneath the Au deposition is still visible, we observe an Au nanocage with a hollow interior after 10 h (Fig. 3c). At this stage, a continuous yet porous Au layer resembling the original cubic shape of the template has formed. The energy-dispersive X-ray spectroscopy (EDX) maps in the insets of Fig. 3 confirm the gradual replacement of Cu_2_O by Au. However, in the SEM images, the interior of the particles is not visible and the mechanism underlying the change of the internal shape remains unknown.

**Figure 4:**
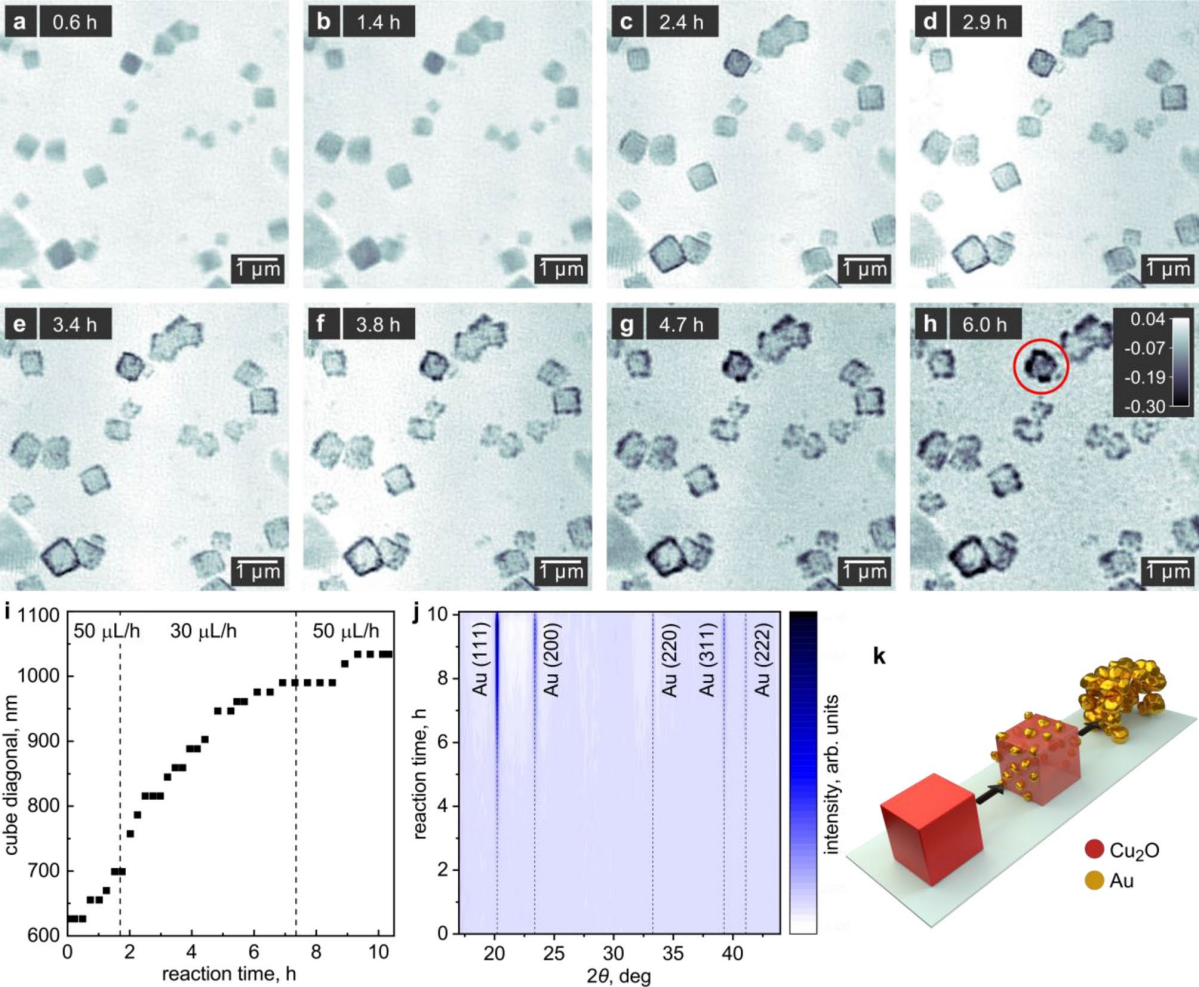
In situ ptychographic images of Au nanocage formation. (**a**–**h**) Ptychographic images reconstructed after different reaction times while dosing the HAuCl_4_ precursor into the reactor. The gray scale indicates the phase shift in radian. (**i**) Size evolution of the nanocube highlighted with a red circle in (**h**). (**j**) Time-resolved XRD patterns obtained alongside the ptychographic images. The reflections of Au become stronger as the replacement proceeds. We observe a significant increase in particle size as Au grains grow at its surface. (**k**) Illustration of the shape evolution during galvanic replacement. While the Cu_2_O in the interior of the nanocages homogeneously dissolved upon oxidation, Au particles nucleate and grow at the surface resulting in porous Au nanocages.

To follow these structural changes during the process, we exploit the strong material contrast available in ptychographic phase images. Figure 4a–h shows an image series taken during the GRR. FRCs indicate a spatial resolution of 82 nm (Supplementary Fig. 7). We can distinguish the first Au particles on the Cu_2_O surface after 2.4 h. There, we observe individual particles on the facets parallel to the image plane, while in the projected view the particles appear as continuous lines along the facets perpendicular to the substrate. As the Au particles grow during the next hours, they start to coalesce and we can distinguish individual, larger Au particles after 3.4 h. The Au particles later form a porous layer covering the entire surface of the template. As exemplarily shown for one of these nanocages in Fig. 4i, the size of that particle increased from about 625 nm to 1050 nm during the GRR. A similar multi-step Au growth mechanism was observed on silica spheres^57^, as well as during the vacuum deposition of Au on polymer^58^ and silicon^59^ surfaces. With our experiment, it is feasible to confirm the mechanism in a more challenging liquid environment.

The reactants of the GRR are sensitive to the X-ray beam, thus, to minimize the beam damage we follow the growth of the Au layer with non-spatially resolved WAXS recorded out of focus (Fig. 4j). For galvanic replacement on metal oxides, both topotactic^41^ and randomly oriented growth^54,55,60^ of the shell material were previously observed. In our case the presence of multiple Au reflections indicates that unlike their Cu_2_O template the newly formed Au particles have no preferred orientation.

In addition to monitoring the growth of the Au shell, the X-ray images enable us to follow the change of the interior of the nanocubes. Within the resolution of our method, we observe that the dissolution of the Cu_2_O template occurs uniformly over the entire volume. The process is illustrated in Fig. 4k.

### Quantification of radiation damage by dose calculation and phase analysis

We observe that repetitive irradiation of the selected field of view leads to X-ray beam-induced deviations from the expected reaction pathway. During the growth as well as during the replacement, additional material deposited onto the substrate at late reaction times (Figs. 2c,d and 4g,h). We parametrize the apparent radiation damage by calculating the surface dose^45^ imparted by the X-ray beam.

**Figure 5:**
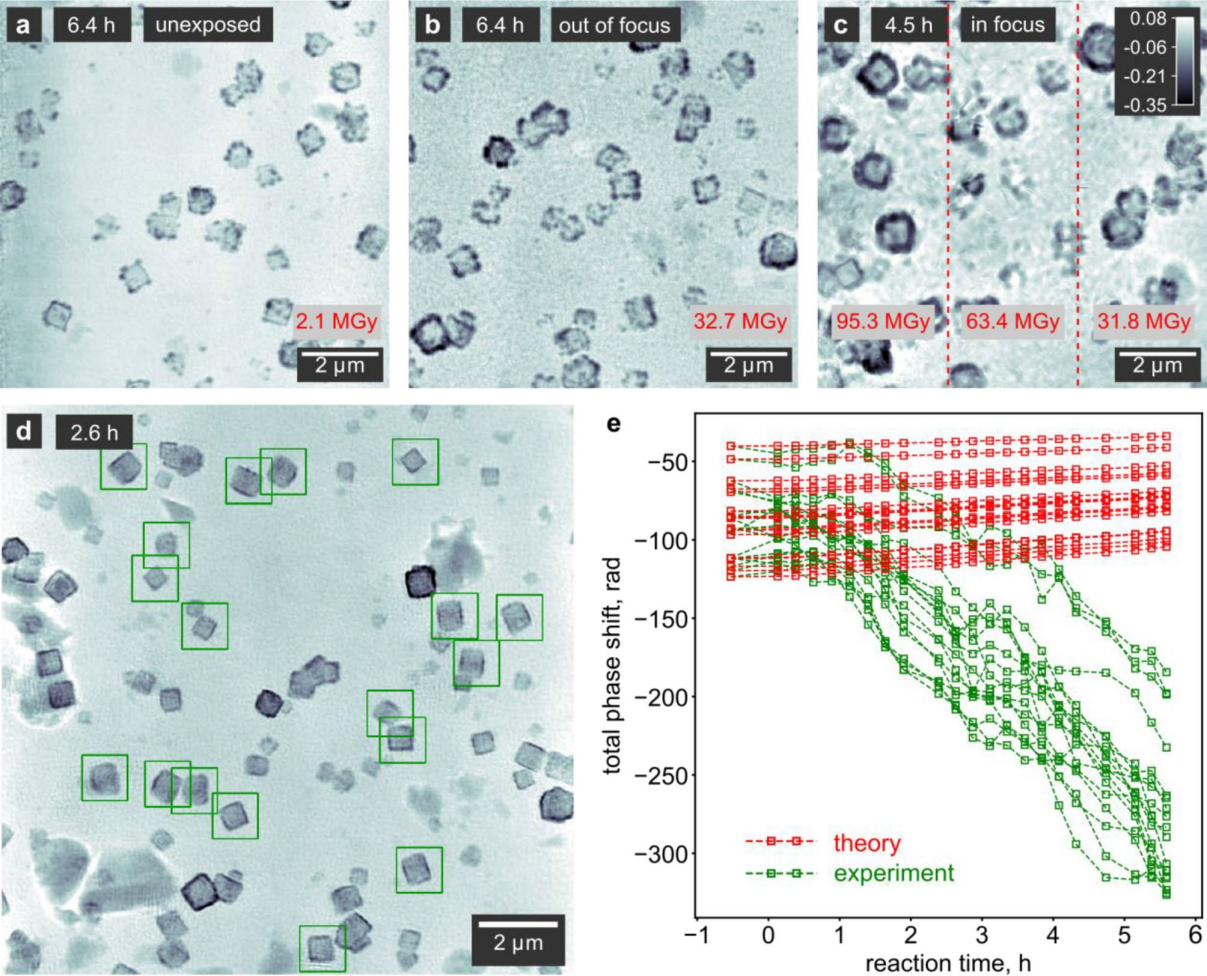
Quantification of radiation damage. (**a**,**b**) In situ ptychographic reconstructions during the GRR obtained at the same reaction time, showing (**a**) a region previously unexposed to X-rays and (**b**) a region continuously exposed for the preceding 6.4 h in the defocus of the X-ray beam. (**c**) Ptychographic reconstruction of a GRR exposed in the X-ray focus with a shifting field of view. The gray scale indicates the phase shift in radian. (**d**) Ptychographic reconstruction highlighting a number of areas around individual particles used for the quantitative phase analysis. The reconstructed pixel size is 14.51 nm. (**e**) Expected evolution of the total phase shift of the particles (red) highlighted in (**d**), assuming a full conversion from Cu_2_O to Au, compared to the measured phase shift (green). We applied a moving average with a window size of 3 data points. A larger negative phase shift indicates more material. Due to the unknown amounts of Cu_2_O and Au in the sample, we can not convert the measured phase shift into an electron count (see Supplementary Note 5 for details).

Figure 5a–c present ptychographic images of GRR recorded under different experimental conditions. The cumulative doses delivered during these measurements are denoted in the lower parts of the images. Figure 5a shows a previously unexposed area of the reactor window 6.4 h after the start of the reaction, while the region in Fig. 5b was continuously imaged over 6.4 h. Both images were recorded using an out-of-focus illumination (compare Supplementary Fig. 8). We observe that a single ptychographic scan with a surface dose of 2.1 MGy in Fig. 5a does not cause visible alterations to the particles. In turn, a cumulative dose of 32.7 MGy in Fig. 5b (for 26 images in total) already results in significantly greater accumulation of Au at the surface of the nanocubes as well as grainy deposition on initially empty areas of the window (see Supplementary Note 3, Supplementary Table 1 and Supplementary Fig. 9 for experimental parameters and details on dose calculation).

To study the effect of dose rate, we imaged the GRR again with an in-focus illumination as shown in Fig. 5c. We observe excessive formation of Au shells on the nanocubes and simultaneous Au deposition on the substrate. The effect is far greater than for the out-of-focus measurement in Fig. 5b. In Fig. 5c, different exposure settings and the use of an in-focus illumination yield a dose of 31.8 MGy per single image acquisition. We shifted the field of view by 3 μm between subsequent images, resulting in 3 subregions (red dashed lines) with respectively decreasing cumulative dose. Although the rightmost subregion received a comparable cumulative dose to the out-of-focus measurement in Fig. 5b, yet the particles in Fig. 5c seem to suffer far more from radiation damage. This may be caused by the almost 50 times greater dose rate per scan point for the measurement in focus (Supplementary Table 1), resulting in respectively higher peak intensities that potentially lead to stronger deviations from the expected reaction pathway.

Furthermore, to follow the additional Au deposition by radiation damage in a quantitative way, we select a number of individual particles in the ptychographic images (green rectangles in Fig. 5d) and plot the total phase shift of each image area vs. reaction time (green data points in Fig. 5e). We correct for a non-zero phase background by creating histograms of the pixel values in the selected areas. The main peak in the histograms corresponds to the average of the background and is offset to zero. Then, the initial per-particle phase shift of the pristine Cu_2_O (first data points in Fig. 5e) corresponds to nanocubes with an edge length between 300 and 450 nm. These values are in good agreement with the nanocube sizes visible in the images, confirming the robustness of our background subtraction method (for details, see Supplementary Note 4 and Supplementary Figs. 10, 11).

For each nanocube, we calculate the amount of Cu_2_O from the phase shift before the start of the GRR using Eq. (3). If we assume no radiation damage and a full conversion to Au according to Eq. (1) at the end of the time series, we can calculate the expected phase shift of the particles in each image (red data points in Fig. 5e). A comparison with the measured phase shift indicates that the beam causes a reduction of additional Au ions that deposit onto the Cu_2_O cubes from approximately 1 h onwards. From Fig. 4a,b we see that this corresponds to the time when the first visual changes to the nanocubes take place. We can thus conclude that the X-ray beam triggers additional Au deposition onto the nanocubes during the entire GRR. From the difference between the theoretical phase shift values and the measurements, we furthermore find an approximately threefold higher amount of Au in the nanocages at the end of the GRR compared to an unexposed reaction.

By imaging a partly unexposed and a partly exposed area within a single ptychographic scan (Supplementary Fig. 12), it is possible to estimate the amount of Au that additionally deposits onto the substrate towards the end of the GRR. The phase difference between the unexposed and exposed areas of the image (green rectangles in Supplementary Fig. 12a) indicates an average thickness of the beam-induced Au deposition of 42 nm.

From a chemical point of view the photolysis of the solvent H_2_O is known to yield hydrogen peroxide (H_2_O_2_), molecular hydrogen (H_2_) as well as highly reactive radial species including hydroxyl radicals (OH^⋅^), hydrogen radicals (H^⋅^) and solvated electrons (e^−^_aq_)^61^. Considering the radiolytic yields at pH 7, the predominantly generated species are OH^⋅^, e^−^_aq_ and to a smaller extent H^⋅^^62^. Both e^−^_aq_ and H^⋅^ are powerful reducing agents which can reduce Au^3+^ ions present in the aqueous HAuCl_4_ solution to elemental Au^0 ^^63^. Under constant irradiation, we can assume that radical species are continuously formed during the experiment. In the absence of Au^3+^ ions radicals undergo back reactions to the molecular products H_2_O_2_ and H_2_^61^. As the Au^3+^ concentrations in the reaction solution steadily increases as a consequence of dosing the HAuCl_4_ precursor into the reactor, it is plausible that a competition of the radical-induced side reaction to the galvanic replacement on Cu_2_O occurs, causing the additional Au deposition.

## Conclusion

We show that multimodal X-ray imaging combining ptychographic phase contrast microscopy with simultaneous scanning diffraction in the nanoprobe is a well-suited tool to observe the formation of nanomaterials directly at the synthesis level in a heated solution. We follow the single-crystalline nature of an individual Cu_2_O nanocube throughout its growth, and we identify the orientation of the cubic crystal lattice. By comparing this to the visual orientation of the nanocube, we confirm the presence of (100) crystallographic planes at the nanocube facets. This simultaneous visualization and crystallographic analysis of an individual nanoparticle reflects in part the information accessible with HR-TEM, but measured in a bulk liquid environment. We then track the galvanic replacement of the nanocubes with HAuCl_4_, yielding Au nanoparticles with various shapes on the surface of Cu_2_O. The phase contrast images allow to clearly distinguish the newly formed layer of Au particles from the interior of the nanocubes. As the Au particles continue to grow, they coalesce and eventually connect to form a porous nanocage. We could furthermore observe that for the nanocubes under study with sizes up to 1 μm, the dissolution of the interior occurs in a homogeneous way. During both, the growth and the GRR, the interaction with the X-ray beam caused the deposition of additional material within the field of view. At the end of the growth stage, small copper-based particles are visible in the background of the images, while during the GRR, reactive species triggered by the beam presumably lead to the additional reduction of Au^3+^ ions beyond the stoichiometry of the GRR. The excess gold deposited onto the nanocages throughout the entire reaction and again, at later stages, additional particles nucleated on the substrate. The phase analysis allows not only to detect but also to quantify the radiation damage based on the known stoichiometry. We observe an approximately threefold higher amount of Au in the nanocages when continuously exposed.

Our work underpins the importance of multimodal X-ray microscopy for the heterogeneous landscape of non-classical formation processes of nanostructures, where many transition pathways from amorphous to crystalline solids remain to be investigated^64–67^. In the future, X-ray fluorescence measurements can provide an additional contrast, enabling to track the composition of amorphous building blocks even before crystallization. Moreover, the progress of fourth generation synchrotron sources with improved coherence at high X-ray energies will allow for an improved spatial resolution and reduced radiation damage. This may reveal more complex morphological changes on size scales below 100 nm currently not accessible with in situ ptychography.

The generality of our approach further encourages its application in related fields. Studying crystallographic and morphological changes during the lithiation and delithiation of lithium ion battery electrodes in an electrolyte^68,69^ or the visualization of unwanted phase changes at the active surface of catalysts in an electrochemical device^70^ may benefit from multimodal in situ visualization.

## Methods

### Synthetic procedure

#### Chemicals

Benzyl alcohol (BnOH) (> 99.8%, anhydrous), Cu(acac)_2_ (99.99%) and HAuCl_4_⋅3H_2_O (≥ 99.9% trace metal basis) were purchased from Sigma-Aldrich, and ethanol (absolute) for washing from VWR. Sodium hydroxide (≥ 99%) was supplied by Carl Roth. All chemicals were used without further purification.

#### *Synthesis of Cu*_*2*_*O nanocubes*

1.72 mL of BnOH was added to 0.03 mmol Cu(acac)_2_ and stirred for 10 min in a glove box under argon atmosphere at room temperature. The solution was transferred to the in situ reactor, sealed, and heated to 138 °C at a rate of 4.5 °C/min. The reaction was stabilized at this temperature, without stirring, for a maximum of 19 h for in situ imaging. All mentioned reaction times are relative to the point when we start heating. For SEM imaging and EDX analysis, the reactor was cooled at a rate of 5.5 °C/min to room temperature. The polyimide windows were rinsed thoroughly with ethanol and dried at 60 °C overnight.

#### Galvanic replacement

Cu_2_O nanocubes prepared in the in situ reactor were used as a starting material. To yield Cu_2_O nanocubes with average edge lengths of 500 nm, the reaction time was decreased to 11 h, the amount of Cu(acac)_2_ was increased to 0.0764 mmol and the volume of BnOH was reduced to 1.6 mL. Further reaction parameters correspond to the values previously described. An empty window was used in the entrance position of the reactor, while the exit window contained nanocubes. 1.4 mL Milli-Q ultrapure water with a resistivity of 18.2 MΩ cm at 25 °C was filled into the reactor. At room temperature, a polypropylene cannula was inserted to dose a 20 mmolar HAuCl_4_ solution with a syringe pump (PHD Ultra, Harvard Apparatus, USA). The pH of the injection solution was adjusted to 7 by adding aqueous NaOH and verified with a pH meter (PH20, VWR, Germany). The injection was started at a rate of 50 μL/h. To achieve a close to uniform reaction speed in the un-stirred reactor, the injection rate was decreased to 30 µL after 1.9 h and re-increased to 50 μL/h after 7.5 h. The reaction time is defined from the start of dosing. After 615 min, the reaction solution was removed to prevent further replacement and the polyimide windows were rinsed thoroughly with Milli-Q ultrapure water and ethanol.

### Ex situ characterization

#### SEM and EDX

Images and maps were measured with a Gemini 1550 SEM (Zeiss, Germany) and an EDX detector Ultim Max 100 (Oxford Instruments, UK) at an acceleration voltage of 10 kV. Samples were coated with 18 nm titanium using a Precision Etching Coating System Model 682 (GATAN, United States) prior to SEM observation and EDX studies.

### In situ reactor

We adopted the in situ rector shown elsewhere^29^. Details about changes to the reactor design for the current study and a schematic are given in Supplementary Note 1 and Supplementary Fig. 1.

### X-ray ptychography and WAXS

#### Beamline setup and data acquisition

Measurements were performed at the Ptychographic Nano-Analytical Microscope PtyNAMi^71^ at the nanoprobe endstation of beamline P06^72^ at PETRA III at DESY in Hamburg, Germany. Stacks of nanofocusing refractive lenses were used to focus the coherent X-ray beam with a photon energy of 15 keV. To collect the small-angle scattering signal for ptychographic imaging, an Eiger X 4 M (Dectris AG, Baden-Daettwil, Switzerland) area detector with a pixel size of 75 × 75 μm² was placed at the end of an evacuated flight tube 3.370 m behind the sample. The WAXS signal was collected using an Eiger X 500K (Dectris AG, Baden-Daettwil, Switzerland) area detector with the same pixel size. It was placed horizontally to the side of the reactor at a distance of 155 mm, with an angle of 40° between beam axis and detector normal. The resulting azimuthal ($\phi$) and polar ($2\theta$) angle coverages were − 27° to 15° and 17° to 45°, respectively. To collect a multimodal data set containing coherent far-field diffraction patterns and spatially resolved WAXS data, the in situ reactor was raster scanned perpendicularly to the beam with its exit window in the focus (Supplementary Fig. 6). For imaging the growth of Cu_2_O nanocubes, a field of view of 10 × 10 μm^2^ with a step size of 100 nm was used with an exposure time of 0.15 s. Each scan took 26 min. For imaging the GRR, both windows were positioned in front of the focus (Supplementary Fig. 8). The field of view was set to 15 × 15 μm^2^ with a step size of 500 nm and an exposure time of 0.5 s, resulting in 15.5 min per scan. The scanning positions were randomly jittered by up to 50% of the step width to avoid artifacts arising from perfect regular sampling in ptychography. A continuous scan mode was used to increase the overlap between adjacent scan points. Furthermore, one reactor window was always positioned out of focus, ensuring enough overlap in at least one object layer. Substantial overlap in one object layer suffices to perform the ptychographic reconstruction.

#### Dose calculation

For each ptychographic scan, the surface dose *D* imparted by the X-ray beam on the specimen was calculated as^45^

2 $$D={\mu }^{{{\prime}}}{\Phi }_{0}E$$

where ${\mu }^{{\prime}}$ is the mass absorption coefficient of the specimen, ${\Phi }_{0}$ is the incident photon fluence, and $E$ is the incident photon energy. Mass absorption coefficients were estimated based on the expected mechanism of the GRR, using the mixture rule^73^ and assuming a linear replacement of Cu_2_O by Au according to Eq. (1). The dose rate per scan point was calculated by substituting the incident fluence ${\Phi }_{0}$ in Eq. (2) with the incident fluence rate per scan point. It does not account for spatial overlap of adjacent scan points and refers to a momentary interaction of X-ray photons with a given region of the specimen within a single exposure. For details on the dose calculation, see Supplementary Note 3.

#### Ptychographic image reconstruction

The diffraction patterns were cropped to 256 × 256 pixels, resulting in a pixel size in the reconstructed images of 14.51 nm. Image reconstruction was done with the extended ptychographic iterative engine (ePIE)^20^. This technique is the subject of patents owned by Phase Focus Ltd. and the University of Sheffield^74–76^. The object was initialized as non-absorbing and non-phase-shifting, and the initial probe was set as a Gaussian probe with a FWHM of 1 μm and a phase curvature of 2.2 mm. The images shown in Figs. 4, 5 were reconstructed with a virtually enlarged probe^77^. We applied multi-slicing to separately reconstruct images of the entrance and exit windows^17^, using the Fresnel nearfield propagator^78^ for wave propagation between slices. The image slice corresponding to the entrance window was initialized first, and the exit window slice was activated after 20 iterations. From iteration 100, position refinement was performed every 20 iterations with a maximum displacement of 44 nm^79^. Reconstructions were run for 1000 iterations. The distance between the slices was initially set to 1 mm. When imaging the growth of nanocubes, it was gradually reduced to adapt to the shrinking distance between the windows of the in situ reactor (for details, see Supplementary Fig. 2).

#### WAXS data analysis

The position of the WAXS detector was calibrated from a diffraction measurement of Au formed at the end of the galvanic replacement reaction using the pyFAI package^80^. Detector images were background-subtracted and azimuthally reshaped with pyFAI, transforming the image data into a cartesian coordinate system of $2\theta$ and $\phi$. Reflections were found by setting a lower threshold to the background-subtracted photon count. Polar-angle histograms of reflection counts showed two distinct peaks corresponding to the offset of the diffraction angle from the two windows (Supplementary Fig. 4). By setting a threshold of $2\theta =19.29^\circ$, the data was separated into sub-sets corresponding to either window. WAXS intensities were assigned to a re-binned raster with a pixel size of 100 nm to represent the expected resolution of the scanning experiment. To this end, refined positions from the ptychographic reconstructions were used. Finally, a logarithmic scale normalized to the maximum signal strength was applied for better visualization of the intensity evolution over the course of a reaction.

### Image analysis

#### Fourier ring correlation

Diffraction patterns of one scan were separated into two data sets with equidistant scan points and reconstructed individually without position refinement, but using the already refined positions from a reconstruction of the full scan. The FRC^51^ was then calculated from the resulting two images. To account for the lower resolution introduced by separating the data set, we estimated the resolution applying the half-bit criterion^52^. For details, see Supplementary Note 2 and Supplementary Fig. 5.

#### Phase analysis and quantification of beam damage

To quantify the phase shift of a given image area, the background was corrected to a shift of 0 rad using a histogram of all pixel values in that area (see Supplementary Note 4 and Supplementary Figs. 10, 11 for details). The corrected phase shift ${\phi }_{\mathrm{corr}}$ of an area containing a single particle was then used to calculate the amount of material as

3 $$n=-\frac{{\phi }_{corr}\lambda {d}^{2}\rho }{2\pi \delta M}$$

where $\lambda$ is the X-ray wavelength, $d$ is the pixel size, $\rho$ and $M$ are the density and the molar mass of the material, respectively, and $1-\delta$ is the real part of the complex refractive index. We used the difference of the refractive index decrement between Cu_2_O and Au, respectively, and the surrounding water resulting in^81^

$${\delta }_{\text{Cu}_{2}\text{O}}=4.1217\cdot {10}^{-6} \;\; \mathrm{and } \;\; {\delta }_{\text{Au}}=1.2126\cdot {10}^{-5}.$$

## Supplementary Information

Supplementary Information.

## Data Availability

The X-ray ptychography, WAXS and SEM data sets generated and analysed during the current study are available in the zenodo repository, 10.5281/zenodo.7014417^82^.
