# Supplementary material for: Multimodal imaging of cubic Cu2O@Au nanocage formation via galvanic replacement using X-ray ptychography and nano diffraction
Source: Sci Rep. 2023 Jan 6;13:318. doi: 10.1038/s41598-022-26877-6 (PMC9823101; doi:10.1038/s41598-022-26877-6)
Supplement: Supplementary file 1 — Supplementary Information. [file 41598_2022_26877_MOESM1_ESM.pdf]

Multimodal imaging of cubic Cu_2O@Au nanocage formation via galvanic replacement using X-ray ptychography and nano diffraction

Authors: Lukas Grote[1,2], Sarah-Alexandra Hussak[1], Leif Albers[1], Karolina Stachnik[1], Federica Mancini[1,4], Martin Seyrich[1,2], Olga Vasylieva[1], Dennis Brückner[1,3], Mikhail Lyubomirskiy[2], Christian G. Schroer[1,2,5], and Dorota Koziej[1,6,*]

Affiliations:
[1] University of Hamburg, Institute for Nanostructure and Solid-State Physics, Center for Hybrid Nanostructures, Luruper Chaussee 149, 22761 Hamburg, Germany
[2] Center for X-ray and Nano Science CXNS, Deutsches Elektronen-Synchrotron DESY, Notkestraße 85, 22607 Hamburg, Germany
[3] Deutsches Elektronen-Synchrotron DESY, Notkestraße 85, 22607 Hamburg, Germany
[4] Institute of Science and Technology for Ceramics (ISTEC), National Research Council (CNR), Via Granarolo 64, 48018 Faenza (RA), Italy
[5] Helmholtz Imaging Platform, Deutsches Elektronen-Synchrotron DESY, Notkestraße 85, 22607 Hamburg, Germany
[6] The Hamburg Center for Ultrafast Imaging, Hamburg, Germany
* Correspondence to: dorota.koziej@physnet.uni-hamburg.de

Electronic Supplementary Information

Supplementary Note 1. In situ reactor

In the present study, we use a modified version of our in situ reactor employed in our previous experiment.[1] Supplementary Figure 1 displays an exploded view of the reactor. A major improvement of the current setup comprises temperature sensors built into the heating elements. This way, the position of the temperature sensors is preserved when re-assembling the reactor, ensuring a steady heating characteristic. We furthermore place rings made from aluminum into the convex Invar plates that push onto the polyimide windows. Making use of the higher thermal expansion of aluminum, the rings have the purpose to stretch the polyimide windows when heated and thereby reduce deformation (compare Supplementary Figure 2).

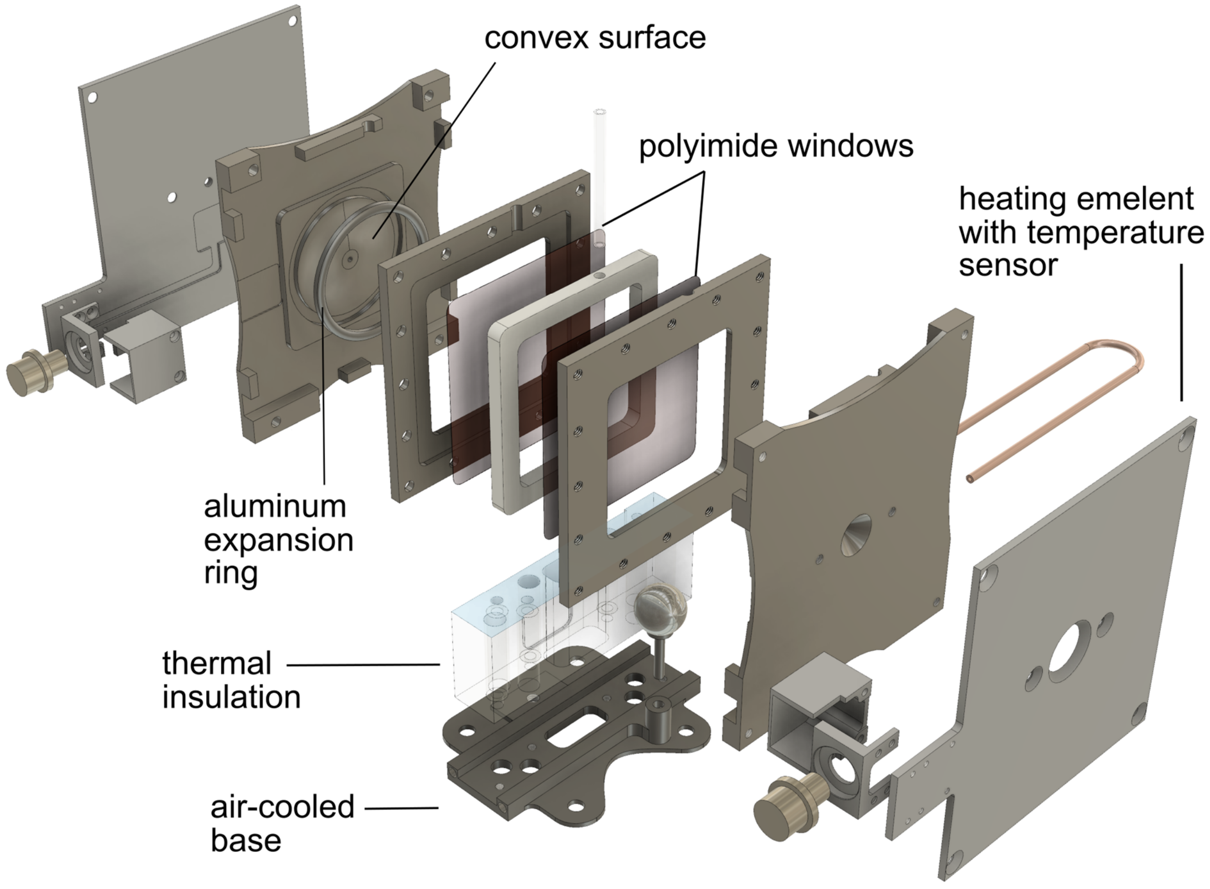

Supplementary Figure 1. Schematic illustration of the in situ reactor.[1] A frame made of PTFE is placed between two polyimide windows and held by two frames made of Invar. The force applied by screwing one of the Invar frames to the other seals the container. A glass capillary allows to fill the container with precursor solution. The container is placed between two Invar plates with a convex surface, reducing the distance between the windows to 1 mm at the center. An expansion ring made of aluminum stretches the polyimide windows when heated. Heat is provided by two flat panel resistive heaters with built-in temperature sensors. A thermal isolation made from Zerodur (Schott Ag, Mainz, Germany) is placed between the heated body and the air-cooled base.

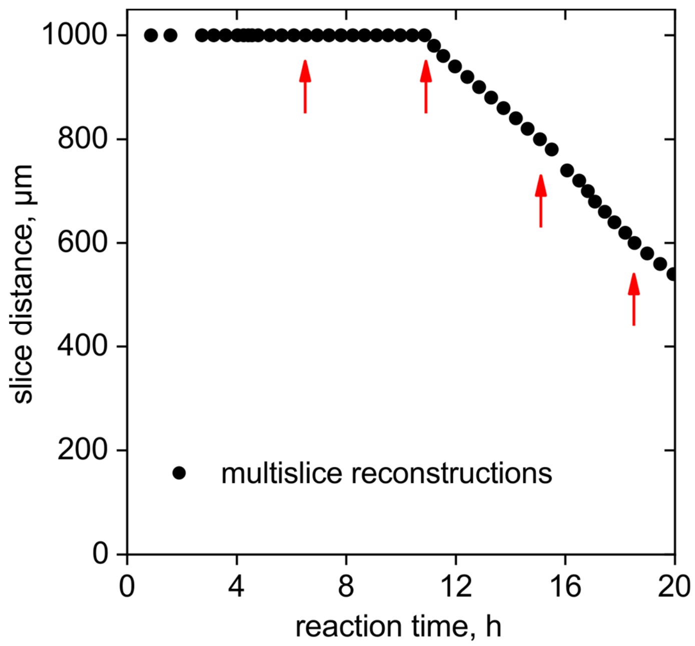

Supplementary Figure 2. Gradual decrease of the window distance. During the growth of Cu_2O nanocubes, the polyimide windows of the reactor slightly deformed, leading to a reduction of the distance between them. We already observed this effect in our previous experiments.[1] The use of aluminum expansion rings in the body of the reactor reduced the effect (compare Supplementary Note 1). While in our previous experiment, the distance decreased below 400 µm after about 16 h reaction time, here the distance is significantly higher after the same time. We determine the distance by visual inspection of the multi-sliced ptychographic reconstructions, as a wrong setting of the slice distance results in reconstruction artefacts.

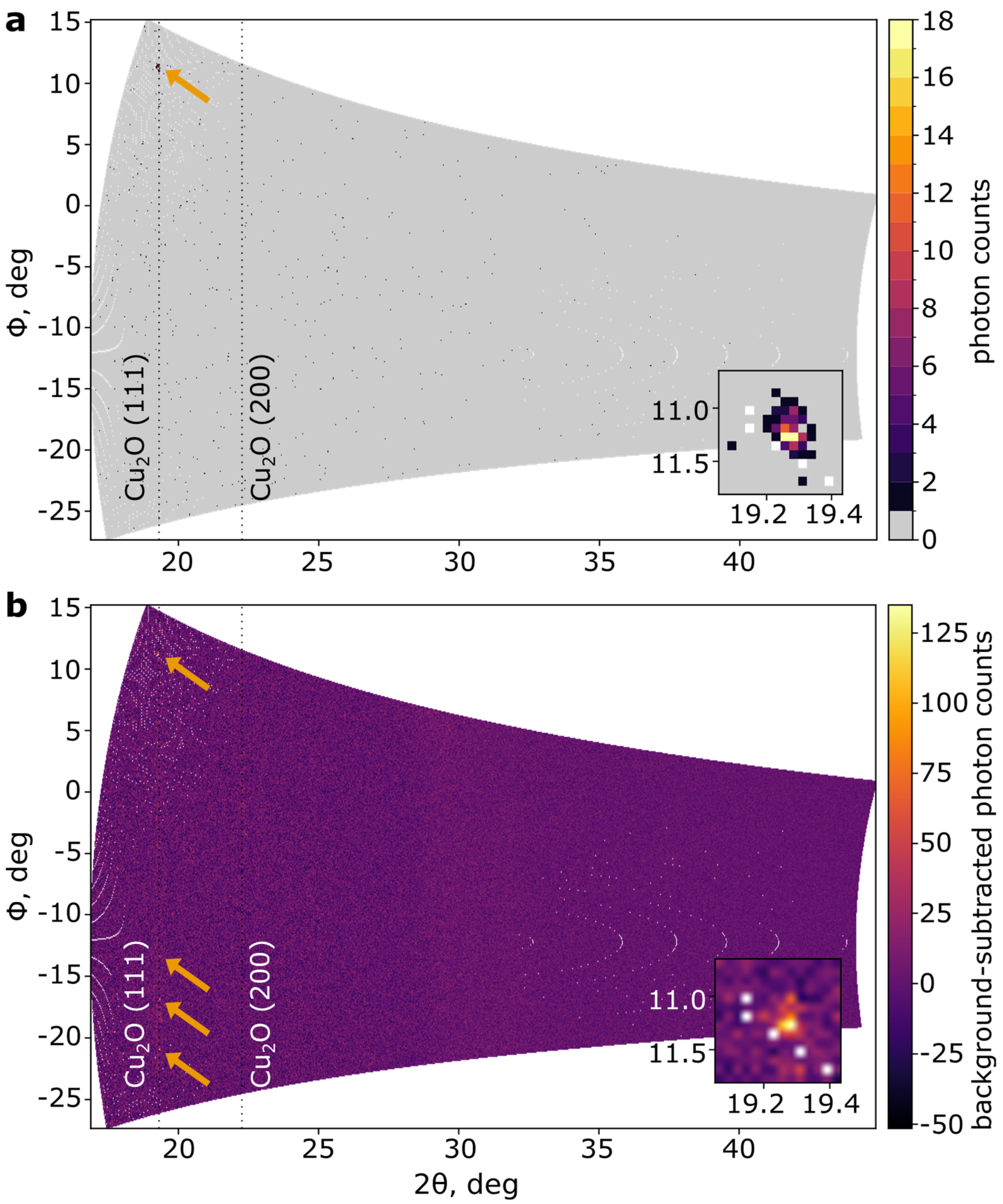

Supplementary Figure 3. Typical WAXS patterns during the growth of Cu_2O nanocubes. The patterns were recorded at 11.5 h reaction time and are coordinate-transformed such that the axes represent the polar scattering angle 2θ and the azimuthal orientation ϕ of the nanocubes. White pixels have no value and originate from the coordinate-transform in combination with the finite resolution of the detector. **a** WAXS pattern of a single scan point with an exposure time of 0.15 s. The pattern was acquired from the interaction of the X-ray beam with the nanocube highlighted with an arrow in Figure 2a. A single Cu_2O (111) reflection can be seen at $2\theta \approx 19.3°$ and ϕ ≈ 11.5° (inset) with a peak intensity of 18 counts. The background counts mostly 0, sometimes 1 and rarely 2 photons in one pixel using a photon counting detector. Note that the orientation of the nanocube of ϕ ≈ 11.5° is slightly different from the average orientation of this particle found in Figure 2g. **b** Sum of all coordinate-transformed diffraction patterns acquired during a full ptychographic scan and background-corrected using a measurement from the reactor without nanocubes. The sum pattern contains less than 5 Cu_2O (111) reflections of individual particles within the field of view.

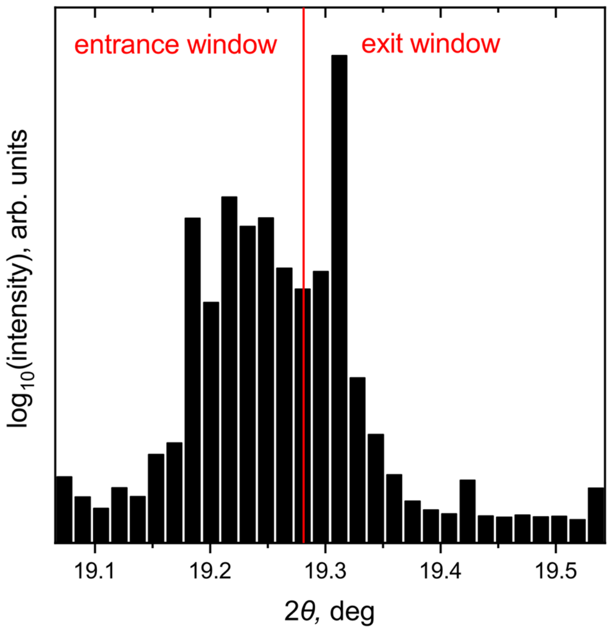

Supplementary Figure 4. **Polar-angle histogram for WAXS separation.** The histogram was obtained from all WAXS intensities of the Cu_2O (111) reflection measured during the growth of nanocubes. Two distinct peaks are visible in the distribution, corresponding to WAXS intensities from the two windows of the reactor. Since these two contributions are not fully separated, WAXS intensities from the respective other window still appear in Figure 2.

Supplementary Note 2. Fourier ring correlation

The Fourier ring correlation[2] (FRC) allows to estimate the resolution of an imaging experiment by correlating two images taken from the same object in Fourier space. Given two Fourier transformed 2D images $\widetilde{I_1}(\boldsymbol{k})$ and $\widetilde{I_2}(\boldsymbol{k})$, the correlation is calculated for rings of $k = |\boldsymbol{k}|$ around the origin of Fourier space:

$$\mathrm{FRC}_{1,2}(k) = \frac{\sum_{|\boldsymbol{k}'|=k}\left[\widetilde{I_1}(\boldsymbol{k}') \cdot \widetilde{I_2}^*(\boldsymbol{k}')\right]}{\sqrt{\sum_{|\boldsymbol{k}'|=k}\left|\widetilde{I_1}(\boldsymbol{k}')\right|^2 \cdot \sum_{|\boldsymbol{k}'|=k}\left|\widetilde{I_2}(\boldsymbol{k}')\right|^2}}$$

Choosing rings in Fourier space results in a 1D correlation which indicates the similarity of the images as a function of the spatial frequency k. For pixel images, no a priori knowledge is required besides the pixel size and the desired width Δk of the rings. The resolution is estimated from the real-space distance corresponding to a certain k_0 where the FRC drops below a cut-off threshold.

Since in our in situ experiment the object is constantly changing, we cannot use two subsequent images for calculating the FRC. Instead, we split the diffraction patterns of a single ptychographic scan into two separate sets with equidistant scan points and reconstruct them individually (Supplementary Figure 5a-c). We then calculate the FRC of the two resulting images (Supplementary Figure 5c). We do not use position refinement during the reconstruction of the split data sets, but we use already refined positions obtained from a previous reconstruction of the full scan. To account for the loss of image quality introduced by separating the data set, we estimated the resolution applying the half-bit criterion.[3]

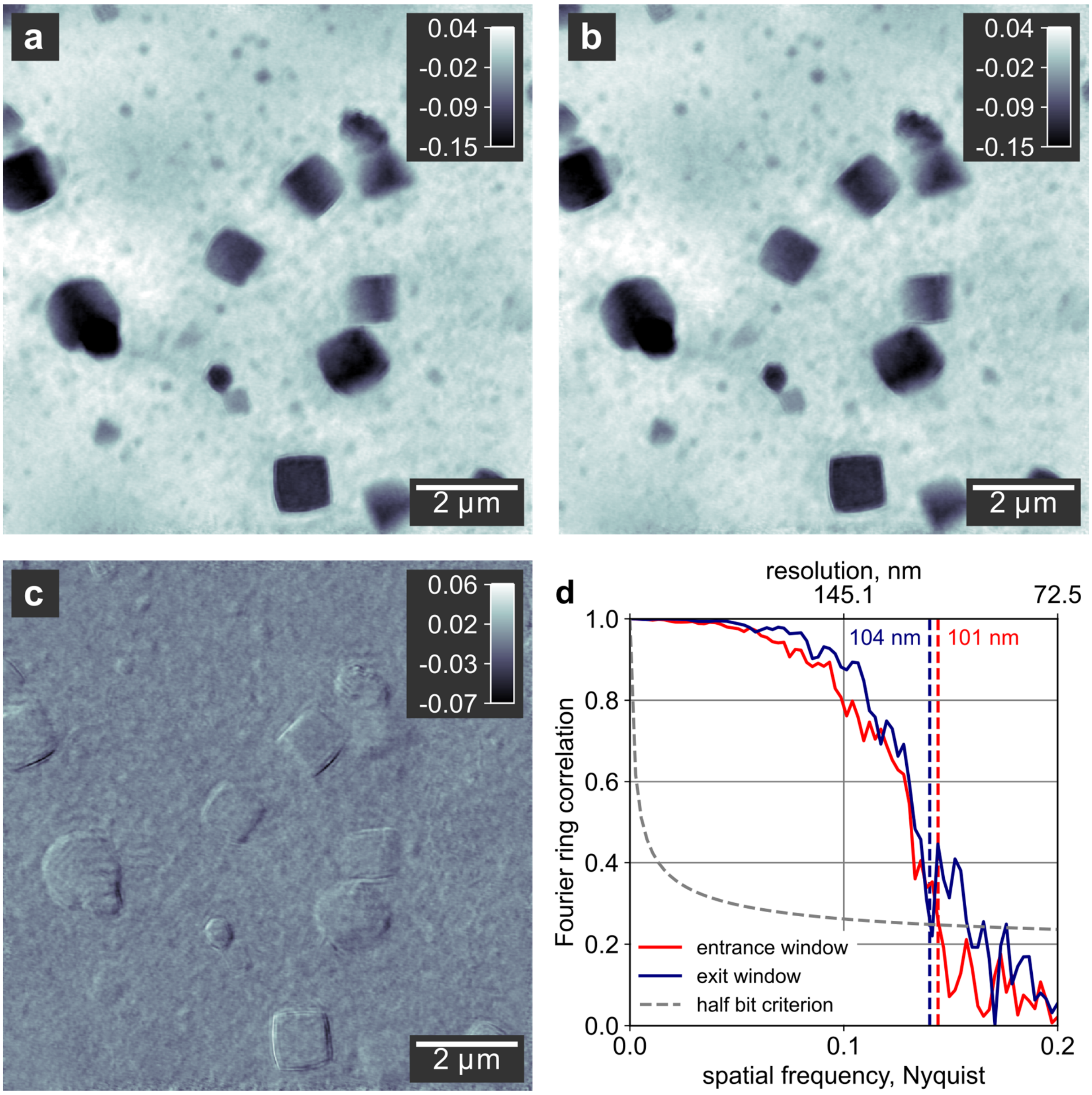

Supplementary Figure 5. Spatial resolution of images during nanocube growth. a-**b** Ptychographic reconstructions of Cu_2O nanocubes taken on the exit window of the reactor. Each image was reconstructed using one half of the scan points of the original data set. The gray scale indicates the phase shift in radians. **c** Difference of the images shown in **a**-**b**. **d** FRC of the images shown in **a**-**b** as well as of respective images taken on the entrance window of the reactor. Using the half-bit criterion, we find a spatial resolutions of 101 nm and 104 nm, respectively.

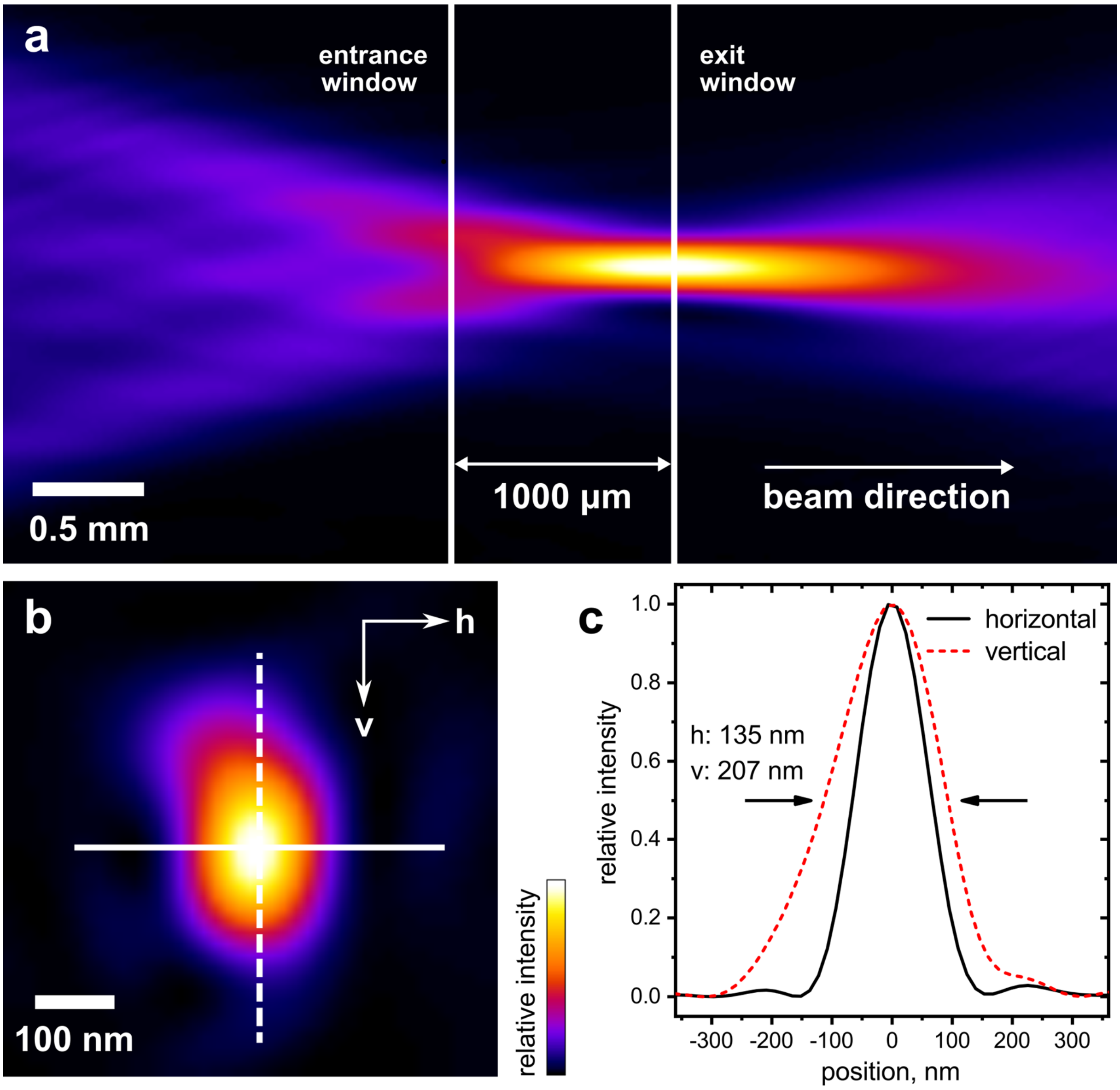

Supplementary Figure 6. Focus characterization and reactor placement for multimodal imaging. a Placement of the reactor windows along the caustic of the focused beam for imaging the growth of Cu_2O nanocubes with combined ptychography and WAXS. The figure shows the horizontal intensity profile. The exit window is placed in the focus. **b** Display of the intensity distribution in the focus. **c** Focus characterization on the exit window denoting the FWHM dimensions in horizontal (black) and vertical (red) directions.

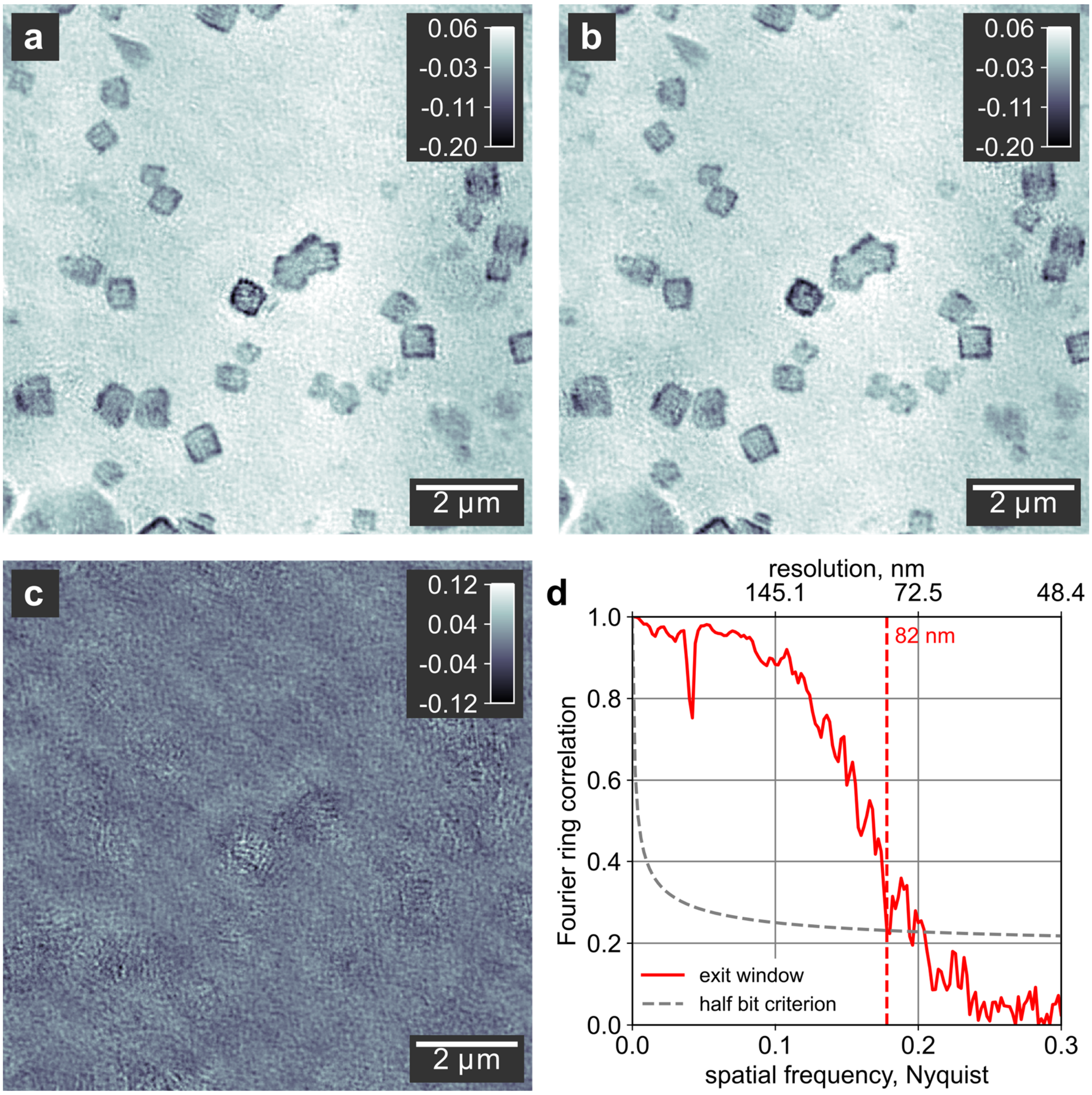

Supplementary Figure 7. Spatial resolution of images during GRR. a-**b** Ptychographic reconstructions taken on the exit window of the reactor. Each image was reconstructed using one half of the scan points of the original data set. The gray scale indicates the phase shift in radians. **c** Difference of the images shown in **a**-**b**. **d** FRCs[2, 3] of the images shown in **a**-**b**. Here, the half-bit criterion indicates a spatial resolutions of 82 nm.

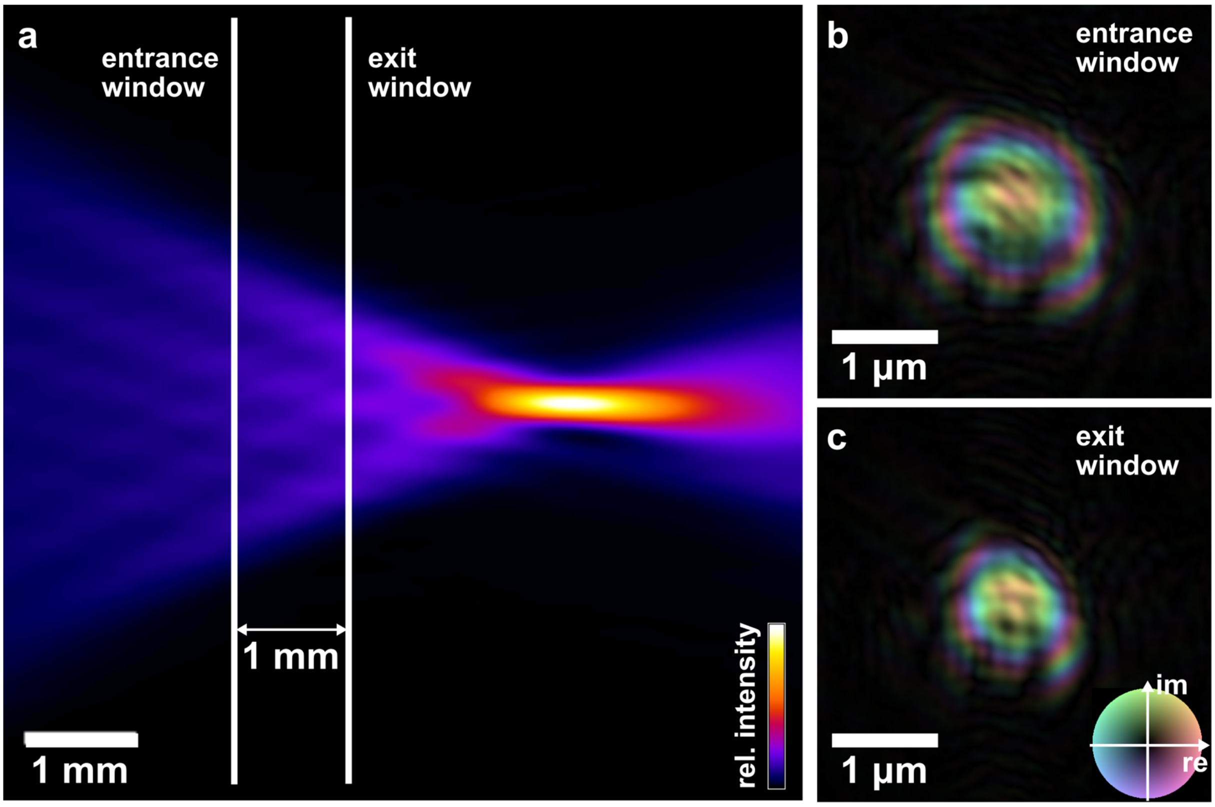

Supplementary Figure 8. Illumination profiles and reactor placement for GRR. a Placement of the reactor windows along the caustic of the focused beam for imaging the GRR, displayed as the horizontal intensity. **b**-**c** Display of the illuminations on the entrance and exit windows, respectively. Brightness represents the amplitude and color represents the phase of the wavefield.

Supplementary Note 3. Quantification of surface dose

In this section we provide detailed information on how we obtained the physical quantities in Equation 2 required to calculate the surface dose and the surface dose rate of the multi-slicing ptychographic measurements reported in the main text.

Incident photon fluence

To determine the incident photon fluence Φ_0, we first identified a scan point with no particles in the X-ray beam path and read out the total number of photons measured in the corresponding far-field diffraction pattern. We then derived the incident photon intensity by correcting the detected intensity for a cumulative X-ray transmission factor of all components of the experimental setup between the sample and the detector. For the reported experiment, the cumulative X-ray transmission factor at a photon energy of 15 keV equaled: 87.8% for the upstream sample slice (entrance window) and 95.5% for the downstream sample slice (exit window). Subsequently, we scaled the corresponding reconstructed illumination function with the previously obtained incident intensity (compare Supplementary Figure 8) and used the scan positions to generate a distribution map of photon intensity accumulated in the scanned area during the measurement. By dividing the total number of photons delivered to the measured region by its surface area, we obtained the incident photon fluence Φ_0. In this way, we accounted for both the illumination size and the substantial overlap between adjacent scan points.

Supplementary Table 1. Detailed experimental parameters of ptychographic measurements shown in Figures 5a-c.

parameter	out of focus (Figs. 5a,b)	in focus (Fig. 5c)
incident photon energy	15.0 keV	15.0 keV
exposure time	0.5 s	0.2 s
scan step size	0.5 µm	0.1 µm
illumination area	1.05 µm^2	0.069 µm^2
flux per scan point	$2.96 \cdot 10^7$ photon/s	$10.0 \cdot 10^7$ photon/s
fluence rate per scan point	$2.26 \cdot 10^7$ photon/µm^2/s	$108.7 \cdot 10^7$ photon/µm^2/s
dose rate per scan point	0.38 MGy/s	18.13 MGy/s

Incident photon fluence rate

The incident photon fluence rate per scan point was obtained by dividing the transmission-corrected photon intensity measured in the far-field detector by the illumination area (Supplementary Table 1). The illumination area was derived from the intensity distribution map of the respective illumination function, counting all pixels above a threshold of 10% maximum intensity. In this way, we obtain a fluence rate per scan point unaffected by overlap between adjacent scan positions.

Mass absorption coefficient

For each ptychographic image acquired during the reaction time window, coefficients s_i of a stoichiometric mixture of $\mathrm{Au}_{s_{Au}}\mathrm{Cu}_{s_{Cu}}\mathrm{O}_{s_O}$ were calculated,[4] according to the expected mechanism of the galvanic replacement reaction (Equation 1). The total atomic mass *A* of the mixture is then given by:

$$A = \sum_i s_i \cdot A_i\,, \ \ \text{for } i = \{Au, Cu, O\}$$

and respective weights w_i:

$$w_i = \frac{s_i A_i}{A}$$

where A_i is the atomic mass of the *i*th element constituting the mixture, $i = \{Au,\ Cu,\ O\}$. Subsequently, the weights were used to derive the mass absorption coefficient of the mixture μ'_{mix}:

$$\mu'_{mix} = \sum_i w_i \mu'_i$$

where μ'_i is the mass absorption coefficient of the *i*th element of the mixture.[5] With an assumption that Au linearly replaces Cu_2O, we utilized this method to calculate mass absorption coefficients for all ptychographic images exhibiting visible structural changes during the reaction. These values were then used to obtain respective surface doses imparted on the sample.

In Supplementary Figure 9, the red curve shows the time evolution of the surface dose delivered within a single projection to the imaged region of the out-of-focus measurement in Fig. 5b in the main text. The outlying point corresponds to a measurement acquired with respectively changed scan and exposure parameters. The dose increases as the replacement reaction progresses (area highlighted in red) due to a gradually changing mass absorption coefficient of the Au-decorated Cu_2O nanocubes. The blue curve in Supplementary Figure 9 shows in turn the cumulative surface dose delivered to the exposed region of the same measurement as a function of reaction time. The monotonic increase of the cumulative dose is modulated by changing composition of the sample during the replacement reaction.

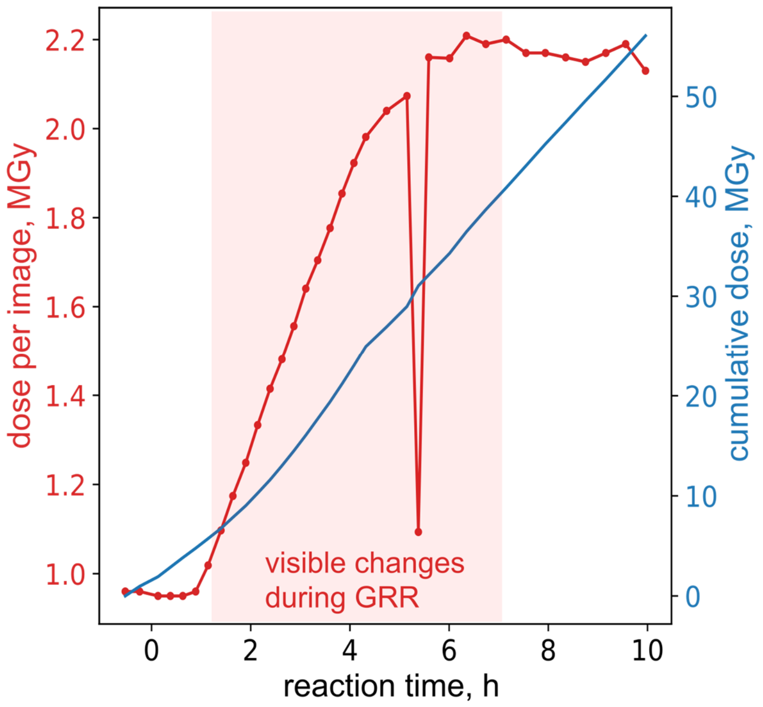

Supplementary Figure 9. Dose estimation for imaging the GRR. Surface dose[6] absorbed by the sample in the exposed region for each image acquisition (red) and cumulative surface dose (blue). The time frame of visible changes to the nanocubes is highlighted. The outlier in the surface dose values corresponds to the acquisition of an overview image with a shorter exposure and a different scan step size.

Supplementary Note 4. Background correction for phase quantification

To be able to quantify the amount of Cu_2O contained within an isolated nanocube in a certain area of a ptychographic image, the phase shift of the background must average to zero. For the in situ images, this is however not always the case. We thus correct for the phase background by creating histograms of all pixel values in the selected image area (Supplementary Figure 10). The histograms typically show one main peak corresponding to the background. We identify the maximum of this peak and apply a phase offset to the image area, shifting the maximum to zero.
An alternative to this approach would be to select an empty area within the background of an image and subtract the average phase shift of this background from the any image area with a particle. However, this method is not as robust due to the occurrence of non-flat phase backgrounds over the entire image area (phase wedges). Supplementary Figure 11 shows a phase analysis of the same particles as selected in Figure 5 but using the average background phase shift within the area highlighted in blue for background correction. While we can observe the same trends in Supplementary Figure 11b and Figure 5e, the phase analysis using the background subtraction method is less stable.

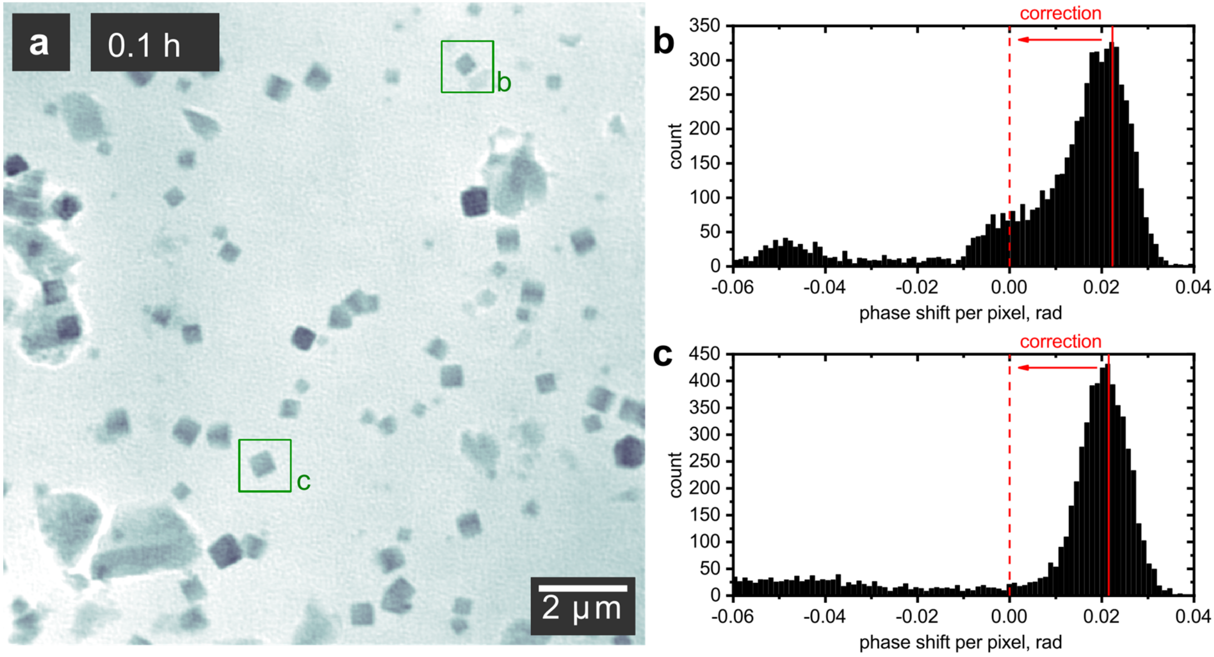

Supplementary Figure 10. Phase background correction. a Ptychographic reconstruction of Cu_2O nanocubes before the start of the GRR. **b**-**c** Histograms of pixel values obtained from the respectively highlighted areas in **a**. Red solid lines mark the maximum of the distribution which is shifted to zero by applying a phase offset.

Supplementary Note 5. Conversion of measured phase shift into electron counts (Figure 5e)

Phase shifts in X-ray imaging are commonly converted into electron counts, which are more intuitive. The conversion is based on the following equation,

$$\phi = \int \delta \, \mathrm{d}z \qquad \text{and} \qquad \delta = \frac{\rho_{at} r_0 \lambda^2}{2\pi}\left(Z + f'(\omega)\right),$$

where ϕ is the measured phase shift along the z dimension of the sample, δ is the refractive index decrement, ρ_{at} is the atomic number density, r_0 is the scattering cross section of the electron, λ is the X-ray wavelength, Z is the atomic number and $f'(\omega)$ is the dispersion correction to the atomic form factor.

In the case of Figure 5e, however, we can not calculate an electron count from the phase shift due to the non-negligible contribution of $f'(\omega)$ for Au. Our measurements were taken at 15 keV, which is close to the L1 and L2 edges of Au at 14.35 and 13.73 keV, respectively. At 15 keV, $f'_{\mathrm{Au}} \approx -4.5$,[5] which is more than 5% of $Z_{\mathrm{Au}} = 79$. Since the amount of Au in the sample is unknown due to the presence of radiation damage, and additionally increases over time, neglecting f′ would lead to a distortion of the plot when scaled in electron counts.

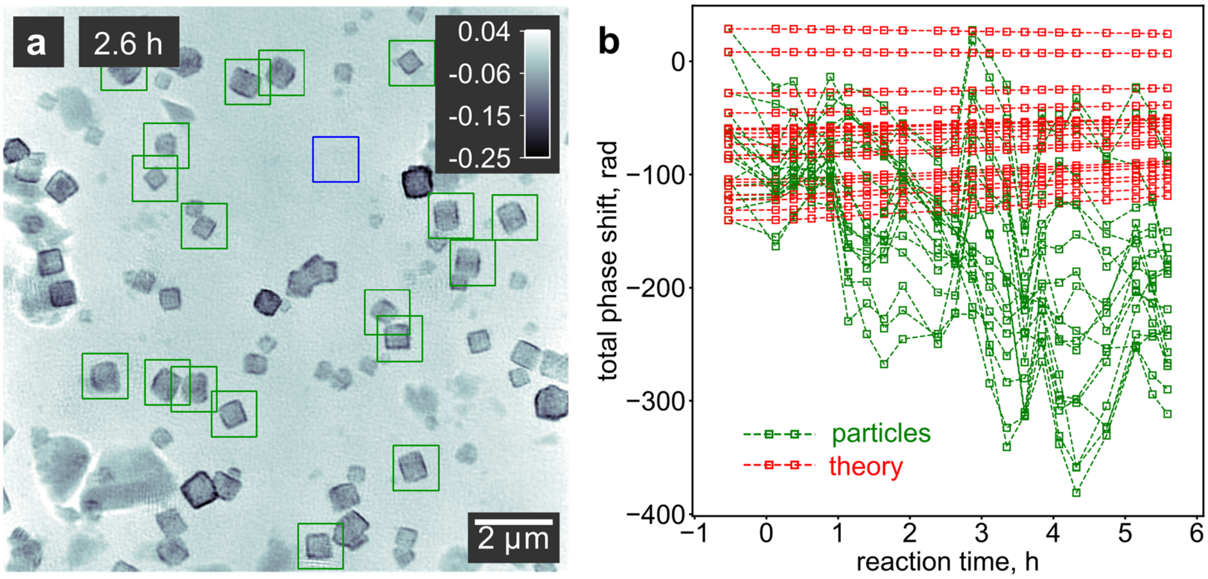

Supplementary Figure 11. Phase analysis with background subtraction method. a Ptychographic reconstruction highlighting a number of areas around individual particles used for the quantitative phase analysis. **b** Expected evolution of the total phase shift of the particles (red) highlighted in **a**, assuming a full conversion from Cu_2O to Au, compared to the measured phase shift (green). In contrast to Figure 5e, here the measured values were background corrected by subtracting the average phase shift in the area highlighted in blue in **a**. We applied a moving average with a window size of 3 data points.

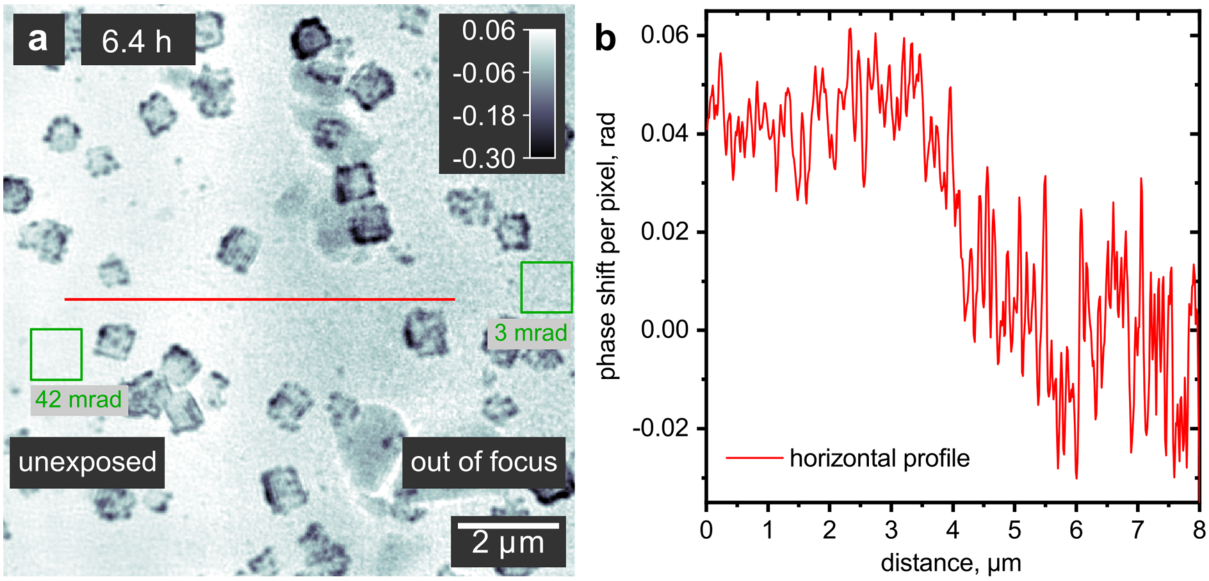

Supplementary Figure 12. Beam-induced Au deposition. **a** Ptychographic reconstruction after 6.4 h of GRR. The left part of the image covers a previously unexposed area, while the right part shows an area continuously exposed during out-of-focus imaging for the entire reaction time. The average phase shift of the two areas highlighted in green is denoted in the image. **b** Phase shift profile along the red line in **a**.

References

1. Grote, L., Seyrich, M., Döhrmann, R., Harouna-Mayer, S. Y., Mancini, F., Kaziukenas, E., Fernandez-Cuesta, I., Zito, C. A., Vasylieva, O., Wittwer, F., Odstrčzil, M., Mogos, N., Landmann, M., Schroer, C. G. & Koziej, D. Imaging Cu2O nanocube hollowing in solution by quantitative in situ X-ray ptychography. *Nat. Commun.*, DOI: 10.1038/s41467-022-32373-2 (2022).
2. Banterle, N., Bui, K. H., Lemke, E. A. & Beck, M. Fourier ring correlation as a resolution criterion for super-resolution microscopy. *J. Struct. Biol.* **183**, 363-367 (2013).
3. van Heel, M. & Schatz, M. Fourier shell correlation threshold criteria. *J. Struct. Biol.* **151**, 250-262 (2005).
4. Jacobsen, C., *X-Ray Microscopy*, Cambridge University Press, Cambridge, 2020.
5. Henke, B. L., Gullikson, E. M. & Davis, J. C. X-ray interactions: photoabsorption, scattering, transmission, and reflection at E=50-30000 eV, Z=1-92. *Atomic Data and Nuclear Data Tables* **54**, 181-342 (1993).
6. Howells, M. R., Beetz, T., Chapman, H. N., Cui, C., Holton, J. M., Jacobsen, C. J., Kirz, J., Lima, E., Marchesini, S., Miao, H., Sayre, D., Shapiro, D. A., Spence, J. C. & Starodub, D. An assessment of the resolution limitation due to radiation-damage in x-ray diffraction microscopy. *J. Electron Spectros. Relat. Phenomena* **170**, 4-12 (2009).
